# Midwifery and the Diseases of Women, Etc.

**Published:** 1861

**Authors:** 


### MIDWIFERY, AND THE DISEASES OF WOMEN, &c.

Amenorrhœa.—*Neuralgia.*—Pains under the left breast, in the intercostal spaces, or in the temples, are very frequent and distressing symptoms occurring in cases of amenorrhœa. They are most quickly relieved by anodynes, but generally only temporarily. The use of small doses of arsenic (two drops of Fowler's solution thrice a day) will be of more ultimate benefit, although not so immediately effectual. When there is any indication of a rheumatic taint of constitution, very satisfactory results will be obtained by the administration of thirty or forty drops of the tincture of *actea racemosa*, or black snake-root (an American plant,) three or four times a day, in a little water. The most rapid relief can be given by the subcutaneous injection of morphia. In *chronic and obstinate neuralgic headache* a very effectual remedy in my hands has been from half a grain to a grain of sulphate or phosphate of nickel. (Prof. Simpson, p 257.)

Application of the Forceps.—The aphorisms Denman so frequently taught in our medical schools and followed by practitioners, that the forceps must never be applied until the head has rested on the perineum for six hours, or except an ear of the fœtus can be felt, are both bad. There are many cases where such delay would be highly injurious, and it is unnecessary to feel the ear of the fœtus. (Dr. A. B. Granville, p. 307.)

Artificial Dilatation of the Os Uteri.—When the os is rigid and contracted in the first stage of labour, and turned towards the rectum, instead of downwards and forwards, first influence the patient gently with chloroform in the following manner: Pour a little chloroform into a tea-cup and hold the edge of the cup to the lower lip of the patient, bringing the open vessel a little over the mouth, but not so as to prevent the free ingress of air both into the mouth and nose; the nurse can manage this whilst you watch the effects. Now introduce the forefinger into the rigid os uteri, and, while the patient is under the influence of the chloroform, gently dilate the part by very quietly, during each pain, pulling the anterior lip towards the pubes. You may keep the patient gently influenced by the chloroform till the os has lost all its rigidity and has become dilated. Now leave off the chloroform till the tough perineum has to be dilated, when you may use it a little again. You will thus save hours of misery and do good to your patient. If the chloroform act too much, cempound spirit of ammonia either taken or breathed is an almost instantaneous antidota. (Dr. Braithwaite, p. 210.)

Cæsarean Section.—If the operation be done with resolution and rapidity, no great alarm need result if we find the placenta attached to the anterior part of the uterus. A sudden gush of blood will follow the incision into it, but the operation must be nevertheless proceeded with, and the placenta be extracted first, as rapidly as is consistent with doing it well. (Dr. J. Edmonds, p. 219.)

Cancer of the Breast.—Sulphate of zinc is one of the best tonics which can be given; somtimes the pain is alleviated, and frequent improvement of the general health follows its prolonged use. (Mr. H. George, p. 260.)

Catamenial Acne.—In some persons a crop of acne appears if the proper menstrual flow is at all checked. In many, this eruption disappears if the flow be restored, but in some it still remains persistent. If, in these cases, you apply the oil or butter of antimony with a brush, very thinly, and neutralize it quickly with tho bicarbonate of soda, the eruption will frequently disappear very speedily; and thus used, the preparation does not cause any pain, nor does it exercise any caustic action. Citrine and mercurial ointments are also very good applications. If one part of the ordinary tincture of iodine be mixed with two parts of the milder aquæ ammoniæ, and allowed to stand forty or fifty hours, the mixture, which is at first brown, becomes quite clear and colorless. As it possesses all the activity of the tincture of iodine, and is yet colorless, it forms one of the best preparations for external application. It may be used to advantage in cases of acne. (Prof. Simpson, p 258.)

CHRONIC MAMMARY ABSCESS.—These cases are sometimes very difficult to cure. It has been recommended to pass a seton across them in a perpendicular direction; but milder means may be employed with success. Apply Scott's ointment to the breast, spread on lint, then place tightly over this strips of plaster an inch and a half in breadth, and bandage the whole carefully. (Mr. W. Coulson, p. 255.)

DEFICIENT INVOLUTION OF THE UTERUS AFTER LABOR.—*Bromide of Potassium.*—Sometimes the uterus does not undergo the normal retrograde metamorphosis after labor, and remains large and heavy, the fibre cells being loaded with fatty particles. The uterus is felt large, soft, and heavy, and there is a feeling of weight and discomfort in the pelvis. In many cases, the abstraction of a small amount of blood from the vaginial portion of the uterus, or the perineum, is called for, and is more especially beneficial in those cases where there lingers any degree of congestion or of inflammatory action in the uterus. The most efficacious of absorbent remedies are the iodide and bromide of potassium. The latter is preferable, however, as it does not produce that marasmus so often caused by prolonged use of the former. The bromide, moreover, acts as a special sedative on the reproductive organs. The dose should be about six grains, three times a day, and it may be continued with iron or other tonics, if anæmia or atony co-exist. Good diet and other hygienic means must be employod. (Prof. Simpson, p. 224.)

FIBROUS TUMOR OF UTERUS.—Intra-uterine fibrous tumors may be destroyed by gouging out a portion of their tissue. The vitality of the growth is so low, that the remaining portion gradually disintegrates, and comes away as discharge. As a preliminary step, it is better to incise the os and cervix uteri. The best instruments to use are those of Mr. Harper, for which see wood-cuts and description at p. 245. (Mr. I. B. Rrown, p. 242.)

Inflation of the Lungs of Infants.—*New Instrument.*—Dr. Wilson, of Glasgow, has invented an instrument for the purpose of inflating the lungs of infants born in an asphyxiated state. It consists of a small india-rubber ball, to which is attached a slightly-curved german-silver tube about six inches long, and having two openings a short distance from the point, and a larger one almost an inch from the attachment of the tube to the ball. The tube is introuced into the larynx, and the larger opening being closed by the finger, the ball is compressed and air forced into the larynx; the finger must be removed during the expansion of the ball that fresh air may enter by the larger aperture. The insufflation of the lungs must be gently and slowly performed, and the chest may be slightly compressed after each inflation. If there is much lividity, allow a drachm or two of blood to escape from the divided vessels of the cord. The instrument may be obtained from Mr. Hillard, surgical instrument maker, Glasgow. (Dr. J. G. Wilson, p 261.)

Ovarian Tumors.—After tapping, apply constant and regular pressure, this much retards the refilling of the cyst. This treatment is only applicable in the monocystic forms of the disease. (Mr. I. B. Brown, p. 247.)

Ovariotomy.—If you think ovariotomy advisable in a case, do not delay it, for after the abdomen has been disturbed by repeated tappings, and the constitution drained by the constant refilling of the cyst, the mortality is very much increased. (Mr. I. B. Brown, p. 248.)

When should the clamp be used, and when the ligature, in securing the pedicle? This depends entirely upon the length of the pedicle; if it is pretty long the clamp is much preferable. If short, we are obliged to use the ligature, letting the stumy of the pedicle return within the abdomen; when this is done the mortality is much greater. (Mr. I. B. Brown, p. 247.)

Sixteen per cent. of the fatal cases die from bleeding from the pedicle. A most excellent plan, and one which renders the use of the clamp unnecessary, is to cut through the bottom portion of the tumor, instead of the pedicle, leaving a portion about the size of a small band, attached; this must be retained outside the abdomen. All risk of serious hemorrhage is avoided. (Dr. Tanner, p. 249.)

The metallic sutures or hare-lip pins, used to close the abdominal wound, must be passed through the peritoneal edges, as well as through the integuments and muscle; otherwise the peritoneum will retract, and a raw surface of considerable breadth will be left exposed to the general peritoneal cavity. (Mr. T. S. Wells, p. 250.)

Besides the precaution of fastening the pedicle externally, and making the smallest external incision compatible with the passage of the tumor, other essentials for success are:—1. To pass silver pins so deeply through the abdominal parietes so as to include the peritoneum. Union by the first intention of the deep parts of the wound is thus secured, and the accidental entrance of any pus into the abdominal cavity is prevented. 2. Nutriment and stimulants must be very freely administered per rectum, when the patient is unwilling or unable to swallow them; they are capable of absorption into the system to a most remarkable extent. (Dr. L. Roberts, p. 250.)

Placenta.—*Removal of, after Labor.*—It is usual to wait for a pain or to be able to feel the insertion of the cord into the placenta, before attempting to remove it. But pains may mislead, and may arise from other causes than constructions of the uterus, and the feeling for the insertion of the cord is a very questionable proof that the placenta has become detached from the uterus. The great objection, however, is, that frequent and painful examinations are sometimes, and indeed often, necessary. The cord after the birth of the child will be found in a state of flaccidity; in a short time, however, it again becomes turgid with blood, which state again passes into one of flaccidity; and this occurs so regularly that this flaccid state following the turgidity constitutes a sign of detachment, which may almost invariably be relied on. (Mr. J. Clay, p. 223.)

Polypus of the Rectum in Children.—Our attention is again called to this affection by a case occurring at Guy's Hospital. The polypus had been overlooked completely by several medical men, who evidently had not read the able article written on the subject by Mr. Bryant last year (*Retrospect*, vol. xli., p. 255.) This affection is most likely to be mistaken for piles, or simple prolapsus of the rectum. There is always hemorrhage from the bowel. The polypus may be broken off by traction, or removed by ligature. (Mr. T. Bryant, p. 263.)

Position in Labor.—In cases of lingering and protracted labor, it is often a good plan to let the woman sit upright upon two chairs fastened to each other *in front* by tape or other strong material, and the *backs* separated one and a-half feet, as may be necessary. The patient during a pain can take hold of the bed-post, and the accoucheur can from time to time ascertain the progress of the case. The patient must be removed to bed when the head is nearly born. In all cases in which this proceeding is adopted, the head should be within the pelvic inlet, and the os uteri dilated fully one half; and further, the failure of the pains in the horizontal position must be first evidenced. (Mr. R. Hardy, p. 301.)

PREGNANCY.—*Operations during.*—If it becomes necessary to perform any operations on a pregnant woman, do not hesitate to perform it; but if it can be delayed till the labour is over, defer it. Some of the most serious operations, as tracheotomy, operations on varicose veins, and removal of condylomata about the anus and vagina, have been performed without any untoward result whatever. (Mr. Shaw, p. 241.)

PREMATURE LABOUR.—*Induction of.*—Pass the tip of the fore-finger of the left hand within the os uteri, and slightly dilate it, pulling the neck down a little, and passing the finger round a little inside. This process, if not accomplished easily at first, may be done more successfully upon a second attempt. Upon each occasion some pains will ensue, and ultimately labour will come on, when an elastic tube may be passed up between the walls of the uterus and the membranes, and a little cold water injected. If any resistance is experienced in introducing it, the tube must be withdrawn and reinserted in another direction. Labour will now proceed naturally. (Mr. H. Sames, p. 217.)

PROLAPSUS ANI OF CHILDREN.—Those surgeons who like to hazard such a mode of treatment, may follow the plan pursued lately in a case of this nature by M. Foucher. This gentleman injected just external to the anus, ten drops of a very weak solution of sulphate of strychnia (twenty centigrammes to twenty grammes of water,) using a Wood's subcutaneous injection syringe. "The cure was immediate." The principle, of course, is the direct action of the salt upon the fibres of the sphincter. (M. Foucher, p. 263.)

PRURIGO PUDENDUM.—In cases of follicular inflammation of the labia, in ecezama, and prurigo pudendum, carefully rub over the diseased portions of skin or mucous membrane a piece of cotton-wool soaked in a solution of nitrate of silver.—This may be continued two or three minutes at a time, and be repeated every day or two.—(Dr. J. Tilt, p. 254.)

PUERPERAL INSANITY.—*Its connexion with Albuminuria.*—It is a curious fact, unknown by the majority of the profession, that in a large proportion of cases of puerperal insanity, albuminuria precedes and attends the first access of the disease.—It, however, generally passes off with extreme rapidity, often in two or three days. Hence it has been so rarely found, on examination, that no connexion between albuminuria and puerperal insanity has hitherto been recognised. When the insanity recurs in the form of successive attacks, each attack will be found connected with a fresh appearance of albumen. If the doctrine of Frerichs be right, that the urea retained, as it always is in cases of albuminuria, is not injurious *per se*, but only from its decomposition into carbonate of ammonia, (albuminuria with, of course, uræmia) causing two other diverse and separate affections—puerperal convulsions and puerperal insanity; the former being caused when carbonate of ammonia results from the change in the urea, and puerperal insanity when some other unknown alkaloid product results. (Mr. Calvert has lately shown that various unknown alkaloids, are formed during animal decomposition.) (Prof. Simpson, p. 235.)

Puerperal Mania.—If, in the onset of a case of puerperal mania, we can induce sleep, the probability is that the patient will wake up tolerably well, or at any rate, the case becomes much more hopeful. The best remedy is opium, but it must be given in doses of two or three grains. If the patient, cannot or will not swallow, you may succeed in introducing the drug in the form of a suppository into the rectum. One or two grains of morphia are required when then the drug is administered in this form. In some cases sleep may be procured by means of ether or chloroform, and the patient thus anæthetised has continued to sleep on and has awaked up quite well.—(Prof. Simpson, p. 233.)

Retroversion of the Gravid Uterus.—In reducing a retroverted gravid uterus it is very important not to attempt to push directly upward at first, as the promontory of the sacrum is in the way. The womb must be placed as much as possible in the right oblique diameter of the pelvis, by drawing the os and cervix towards the left acetabulum, and moving the fundus as much as possible towards the sacro-iliac syncondrosis. It is also well before commencing much manipulation, to introduce compressed sponge into the vagina, to dilate that canal for the more ready introduction of the hand, and to exercise gentle and steady pressure on the uetrus. (Dr. T. Skinner, p. 213.)

Sore Nipples.—A mixture of equal parts of brandy and glycerine forms an excellent application. (Dr. W. Frazer, p. 260.)

Suppression of the Secretion of Milk.—Moisten a flannel with saturated solution of camphor in glycerine, and apply it over the breast. This will often check the secretion of milk very quickly. (Dr. Harriss, p. 260.)

Vaginitis.—Inject a solution of Nitrate of silver (two scruples to the ounce.) This acts equally well whether the vaginitis be the result of uterine catarrh, or occurs spontaneously. It is most conveniently done if a small glass speculum be inserted first; as the speculum is withdrawn the solution follows it and may be received in a cup. (Dr. E. J. Tilt, p. 254.)

Vesico-Vaginal Fistula.—Do not operate in these cases when the woman is suckling. Also avoid operating on a ruptured perineum at this period. The patient is more liable to pyæmia at such times. [Mr. I. B. Brown, p. 240.]

Mr. Buxton Hilliard, surgical instrument maker to the Glasgow Royal Infirmary, has invented a set of instruments which we think will prove extremely useful in the tedious operation for the cure of vesico-vaginal fistula. The speculum is one which, preserving the form of the vaginal, expands so as probably to give a better view of the parts than any speculum previously in use. The edges of the fistula are seized by an instrument called a "fistula clamp," which is so contrived that the edge of the fistula, when seized by it, is elevated above the surrounding parts, and only requires shaving off with a slightly curved knife. When Bozeman's knives are used, there is often much uncertainty whether the whole of the edges have been effectually pared or not; when this instrument is employed there can be no doubt about it. The tubular needle invented by Mr. Price is the best instrument for passing the metallic sutures through the edges of the wound. The ends of the ligature are brought together and twisted by means of another instrument, and an oval metallic plate applied, differing from Bozeman's in having nipples attached to it, instead of loose pellets being employed. Much trouble is thus saved, otherwise caused in passing each pellet separately. These instruments will be better understood by referring to the original article and the engravings at page 238.—*Braithwaite.*

